# Evaluation of adverse drug reactions in cancer-related settings using diverse real-world data

**DOI:** 10.1186/s40780-026-00587-x

**Published:** 2026-05-23

**Authors:** Ryo Inose

**Affiliations:** Laboratory of Clinical Pharmacoepidemiology, Kyoto Pharmaceutical University, 5 Misasagi Nakauchi-cho, Yamashina-ku, Kyoto, 607-8414 Japan

**Keywords:** Real-world data, Adverse drug reactions, Cancer, Pharmacovigilance, Electronic medical records, Spontaneous reporting systems, Medical claims databases, Diagnosis procedure combination

## Abstract

The evaluation of adverse drug reactions in cancer-related settings represents a complex clinical challenge that extends beyond the toxicities associated with anticancer therapies, including drug-induced malignancies and treatment-related complications, in heterogeneous patient populations. Since randomized controlled trials are conducted in highly selected populations, their findings are not always directly generalizable to routine clinical practice. In this context, real-world data (RWD) have become an important resource for evaluating drug safety and clinical outcomes in real-world clinical practice. This review provides an overview of RWD-based approaches for evaluating adverse drug reactions in cancer-related settings, with a particular focus on how different data sources can be used to address clinically relevant safety questions. Electronic medical records enable the detailed evaluation of patient-level clinical information and have been used to identify clinical and pharmacokinetic factors associated with adverse drug reactions. Spontaneous adverse event reporting databases are particularly useful for detecting rare and unexpected adverse events, including drug-induced malignancies. Medical claims databases enable large-scale longitudinal analyses and evaluation of the incidence and time-to-onset of adverse drug reactions. Additionally, the Diagnosis Procedure Combination database provides detailed inpatient information and has been used to evaluate treatment-related complications in routine clinical practice. As each type of RWD has distinct strengths and limitations, the choice of an appropriate data source should be guided by a specific research objective. Selecting the most suitable RWD source according to the research question is essential for appropriately evaluating adverse drug reactions. This approach enables the assessment of diverse clinically relevant safety issues in cancer-related settings and supports the generation of evidence that reflects routine clinical practice.

## Background

Cancer is the leading cause of death worldwide, with approximately 10 million deaths reported in 2020 [1]. In Japan, cancer also represents a major public health burden; in 2024, approximately 400,000 deaths were attributed to cancer [2]. Current cancer treatment strategies are based on three major modalities: surgery, radiotherapy, and chemotherapy [3]. These modalities can be applied alone or in combination, depending on the type and stage of the disease.

In recent years, a wide range of anticancer agents, including cytotoxic chemotherapy, molecularly targeted therapy, and immune checkpoint inhibitors, have been introduced into clinical practice [4]. However, adverse drug reactions associated with cancer treatment remain a major challenge in cancer-related settings as they often lead to dose reduction, treatment interruption, or discontinuation of anticancer therapy, which has been associated with poorer clinical outcomes [5]. Despite the development of supportive care strategies and toxicity management guidelines [6–8], many aspects of the relationships between anticancer therapies and their adverse drug reactions remain insufficiently understood, underscoring the need for further clinical research.

Nevertheless, clinical challenges in cancer-related settings extend beyond the management of adverse drug reactions during anticancer therapy. One such example is drug-induced malignancy [9]. Although relatively rare, drug-induced malignancies can have serious implications for patient prognosis and, therefore, represent an important clinical concern. In addition, a substantial proportion of patients with cancer are older adults and therefore represent a heterogeneous population with respect to clinical characteristics, often presenting with poor performance status, organ dysfunction, and multiple comorbidities [10]. Such patients are commonly excluded from randomized controlled trials [11, 12]. Consequently, the findings obtained from randomized controlled trials may not always be directly generalizable to patients encountered in routine clinical practice. Therefore, the validation of findings from randomized controlled trials in real-world clinical practice is an important clinical challenge in cancer-related settings.

Real-world data (RWD) [13] refers to data routinely collected during everyday clinical practice and encompasses a wide range of data sources, including electronic medical records (EMR), spontaneous adverse event reporting, medical claims, and Diagnosis Procedure Combination (DPC) databases. In this review, EMR data primarily refers to data obtained from a single or a limited number of healthcare institutions rather than large-scale integrated EMR databases or nationwide clinical data repositories. Unlike randomized controlled trials, which are conducted in highly selected and controlled patient populations [14], RWD enables the evaluation of treatment outcomes and adverse drug reactions in heterogeneous patient populations that more closely reflect routine clinical practice.

Because each type of RWD has distinct strengths and limitations, the choice of an appropriate data source should be guided by a specific research objective [15]. An overview of these characteristics is provided in Table 1. For example, EMR provides detailed patient-level clinical information [16], spontaneous adverse event reporting databases are particularly useful for detecting rare safety signals [17], and medical claims databases enable large-scale population-based analyses [16]. The DPC is a hospital-based administrative database providing detailed information on inpatient diagnoses, procedures, and treatments [15]. Therefore, selecting the most suitable RWD source according to the research question is essential to appropriately evaluate adverse drug reactions.

**Table 1:** Characteristics and selection of real-world data sources for evaluating adverse drug reactions

Data source	Key characteristics	Strengths	Limitations	Typical research applications
Electronic medical records	Detailed patient-level clinical data, including laboratory values, treatment course, and clinical outcomes	Granular clinical information; assessment of clinical context and pharmacokinetics	Limited sample size; missing data; variability across institutions; potential selection bias; limited generalizability	Identification of clinical and pharmacokinetic risk factors; detailed clinical evaluation of adverse drug reactions
Spontaneous adverse event reporting databases	Large-scale accumulation of reported drug–adverse event pairs	Detection of rare and unexpected adverse events; signal detection	Lack of denominator; reporting bias; limited clinical detail	Detection of potential drug– adverse event associations
Medical claims databases	Longitudinal administrative data covering large populations across institutions	Large-scale population-based analysis; long-term follow-up; evaluation of incidence and temporal patterns	Limited clinical detail; lack of laboratory data; potential misclassification of exposure and outcomes	Estimation of incidence and time-to-onset; analysis of treatment patterns
DPC databases	Hospital-based inpatient administrative data derived from acute care hospitals in Japan, with detailed information on diagnoses, procedures, and treatments	Detailed inpatient treatment information; evaluation of short-term outcomes	Limited to inpatient setting; limited long-term follow-up; limited clinical detail	Evaluation of treatment-related complications; short-term outcomes during hospitalization; evaluation of healthcare interventions

The categories of research applications presented in this table are not mutually exclusive, and a substantial overlap exists among the real-world data sources in their potential applications. Therefore, the selection of a data source should be guided by a specific research objective rather than by a fixed classification because each real-world data type has distinct and complementary strengths and limitations

This review provides an overview of RWD–based approaches for evaluating adverse drug reactions in cancer-related settings, with a particular focus on how different data sources can be used to address clinically relevant safety questions.

## EMR for detailed clinical evaluation of adverse drug reactions

EMR, especially those derived from a single or a limited number of institutions, provide detailed patient-level clinical information, including patient characteristics, treatment courses, laboratory data, and clinical outcomes [16]. A key advantage of EMR data is the availability of granular clinical information, which enables in-depth evaluation of adverse drug reactions that are often difficult to fully characterize in randomized controlled trials. Because of these characteristics, EMR data have been widely used to investigate the risk factors associated with treatment-related adverse drug reactions in patients with malignancies. For example, studies have evaluated the risk of hypomagnesemia associated with cetuximab therapy in patients with head and neck malignancies and identified a history of cisplatin administration as a potential risk factor [18].

EMR data can also be applied to the evaluation of pharmacokinetics and drug exposure in clinical practice because they often include detailed laboratory information such as plasma drug concentrations and other clinical test results. High-dose methotrexate (MTX) therapy is widely used for the treatment of central nervous system lymphoma and is considered a backbone of therapy in current clinical practice [19, 20]. However, sustained elevation in plasma MTX concentrations during high-dose MTX therapy is associated with an increased risk of adverse drug reactions [21]. EMR-based studies have identified the concomitant use of calcium channel blockers as a potential factor associated with delayed MTX elimination [22].

Furthermore, EMR data allow the evaluation of treatment outcomes and treatment intensity in routine clinical practice. Because EMRs record detailed information on treatment schedules, dose reductions, and treatment discontinuation, they can be used to analyze the clinical factors associated with treatment continuation and relative dose intensity (RDI). A previous study reported that early initiation of adjuvant S-1 chemotherapy after pancreatic cancer surgery, as well as the occurrence of grade 3 neutropenia during treatment, may be associated with an improved prognosis [23]. In addition, a previous study examined the differences in the RDI of adjuvant chemotherapy between elderly and nonelderly patients with colorectal cancer [24].

EMR-based studies are also useful for evaluating infection-related clinical issues in patients with malignancies. In particular, patients with hematologic malignancies are highly susceptible to infections owing to disease- and treatment-related immunosuppression [25]. Cytomegalovirus (CMV) infection is a common complication among patients undergoing hematopoietic stem cell transplantation (HSCT) [26]. Compared to ganciclovir, foscarnet causes less myelosuppression [27] and therefore represents an important alternative therapeutic option for the management of CMV infection in HSCT patients. However, acute kidney injury (AKI) occurs frequently during foscarnet therapy [28] and may necessitate modification of concomitant therapies, potentially compromising optimal cancer treatment, thereby potentially compromising optimal cancer treatment. An EMR-based study suggested that prolonged foscarnet therapy may be a clinically relevant risk factor for AKI in patients undergoing HSCT [29], highlighting the importance of prevention and appropriate management of AKI. In addition, in patients with febrile neutropenia with hematologic malignancies, the combination of vancomycin and piperacillin–tazobactam has been reported to be associated with a significantly higher incidence of AKI than vancomycin combined with other antipseudomonal antibiotics [30].

Although EMR data have enabled a wide range of investigations, including the evaluation of adverse drug reactions, pharmacokinetics, treatment intensity, and infection-related clinical issues in patients with malignancies, several limitations remain. EMR-based studies often have relatively limited sample sizes, making it difficult to evaluate rare adverse drug reactions. In addition, missing data, variability in data recordings, and differences in documentation practices across institutions may introduce bias and limit the generalizability of the findings. Therefore, these limitations should be carefully considered when interpreting the results of EMR-based analyses.

## Spontaneous adverse event reporting databases for signal detection of rare adverse events

Spontaneous adverse event reporting databases are an important source of information for evaluating drug safety [17]. Regulatory authorities in many countries have established spontaneous reporting systems as part of their pharmacovigilance activities, and several large databases are currently in operation, including the U.S. Food and Drug Administration Adverse Event Reporting System and the Japanese Adverse Drug Event Report database. These databases accumulate reports of suspected drug-adverse event associations submitted by healthcare professionals and pharmaceutical companies. Using statistical approaches, such as disproportionality analysis, these databases enable the evaluation of whether a particular drug–adverse event combination is reported more frequently than expected compared with other drugs, thereby facilitating the detection of potential safety signals [17].

A key advantage of spontaneous adverse event reporting databases is their ability to detect rare and unexpected adverse events through the large-scale accumulation of case reports [17]. Even when such events are difficult to evaluate in EMR-based studies because of limited sample sizes, spontaneous reporting databases may allow signal detection based on large-scale case aggregation. For example, cardiovascular toxicities associated with immune checkpoint inhibitors have been investigated using spontaneous adverse event reporting databases [31].

Spontaneous reporting databases also play an important role in evaluating adverse events with long latency periods, such as drug-induced malignancies. Since the early 1990s, cases of malignant lymphoma have been reported in patients with rheumatoid arthritis treated with MTX [32], raising concerns regarding MTX-associated lymphoproliferative disorders. Currently, combination therapy with MTX and biological disease-modifying antirheumatic drugs (bDMARDs) is widely recommended for patients with an inadequate response to MTX monotherapy [33, 34]. However, the evidence regarding the association between bDMARDs and malignant lymphoma remains inconsistent, with conflicting results reported across studies [35–38]. Although malignant lymphoma has been a primary focus of concern, the relationship between MTX-based therapy and a broader spectrum of malignancies has not yet been fully elucidated.

Against this background, analyses using spontaneous adverse event reporting databases have been conducted to evaluate the association between combination therapy with MTX and bDMARDs and malignancies in patients with rheumatoid arthritis [39, 40]. These analyses suggest that combination therapy with MTX and bDMARDs may be significantly associated with malignant lymphoma [39]. Furthermore, signals suggesting an association with several solid malignancies, including breast, ovarian, and lung cancers, have been reported [40]. These findings indicate potential associations with rare but clinically important adverse events and highlight the need for further validation through epidemiological studies.

Besides detecting increased reporting signals, spontaneous reporting databases have also been used to investigate so-called “inverse signal” in which a specific drug–adverse event combination is reported relatively less frequently compared with other drugs [41]. Although inverse signals do not indicate protective drug effects or causal relationships, such findings may provide hypothesis-generating observations for future research. For example, studies have identified concomitant medications that show inverse signals for hyperglycemia in patients treated with antipsychotic agents, and subsequent experimental investigations have explored their potential therapeutic implications [42]. Similarly, inverse signals between sodium-glucose cotransporter 2 inhibitors and specific malignancies have been detected [43], and should be cautiously interpreted as hypothesis-generating observations.

Overall, spontaneous adverse event reporting databases play a crucial role in detecting safety signals for rare but clinically significant adverse events and exploring novel drug–adverse event associations. However, these databases have inherent limitations, including a lack of information on the number of exposed patients and the limited availability of detailed clinical data, which makes it difficult to directly estimate incidence rates or establish causal relationships. Therefore, the safety signals identified in spontaneous reporting databases should be further evaluated and validated through epidemiological studies using other types of RWD.

## Medical claims databases for large-scale longitudinal and incidence analyses

Among various sources of RWD, medical claims databases are important resources for large-scale longitudinal studies. This database systematically records healthcare utilization information, including diagnoses, procedures, and prescribed medications, thereby providing comprehensive data that reflect healthcare delivery in routine clinical practice [16].

A key advantage of medical claims databases is the ability to track patients longitudinally across multiple healthcare institutions. As patient records are linked through insurance claims rather than individual hospitals, patients can be followed up, even when they receive care at different medical facilities. This characteristic enables long-term safety evaluations and population-based analyses, which are difficult to conduct using single-institution EMR data.

Because of these strengths, medical claims databases have been widely used to evaluate the incidence of rare adverse drug reactions and the latency period between drug exposure and the onset of adverse drug reactions. Long-term follow-up is essential for adverse drug reactions with long latency periods, such as drug-induced malignancies. Medical claims databases were analyzed to evaluate the association between MTX combined with bDMARDs and the incidence of malignancies in patients with rheumatoid arthritis [44]. These analyses demonstrated that the incidence of malignant lymphoma was significantly elevated in both the MTX monotherapy and MTX–bDMARD combination therapy groups compared with that in the general population. Moreover, the interval between MTX initiation and the onset of malignant lymphoma spans several years, underscoring the importance of long-term follow-up when evaluating drug-induced malignancies. Compared with patient registries, which require substantial resources for establishment and maintenance, medical claims databases allow efficient large-scale analyses at lower cost and within a shorter timeframe, thereby representing an alternative approach for evaluating the malignancy risk associated with drug exposure [44].

Besides safety evaluations, medical claims databases are increasingly being used to investigate treatment patterns and temporal changes in therapeutic strategies in real-world clinical practice. Because these databases capture longitudinal treatment records across large patient populations, they are well-suited for assessing the uptake of newly introduced therapies and shifts in treatment strategies over time. For example, claims-based studies in Japan have evaluated treatment patterns in patients with hormone receptor-positive and human epidermal growth factor receptor 2-negative metastatic breast cancer in real-world clinical practice, demonstrating that treatment patterns are generally consistent with clinical guidelines while varying according to patient characteristics [45]. Such studies provide valuable insights into treatment patterns in routine clinical practice that cannot be fully captured by randomized clinical trials.

Collectively, medical claims databases provide a powerful platform for evaluating the incidence and timing of adverse drug reactions as well as for characterizing treatment patterns in large patient populations. However, because these databases are primarily designed for administrative purposes, they typically lack detailed clinical information such as laboratory values and disease severity. In addition, studies using medical claims databases rely on claims-based definitions to identify clinical outcomes and adverse drug reactions. Therefore, the validity of these definitions should be carefully considered, as misclassification of exposures or outcomes may affect the accuracy of incidence estimates and time-to-onset analyses. Validation of outcome definitions is important to ensure that the study findings are appropriate for the specific research objective.

## DPC databases for inpatient outcomes and short-term complications

The DPC is a large hospital-based administrative database that collects inpatient healthcare information from acute care hospitals across Japan [15]. It systematically records detailed information on hospitalized patients, including diagnoses, procedures, surgeries, and prescribed medications. Because these data were collected from numerous medical institutions participating in the DPC system across Japan, the database reflects real-world inpatient care in acute care hospitals across Japan and has been widely utilized for clinical and epidemiological research.

A key advantage of the DPC database is the availability of detailed information on the treatments, procedures, and medications administered during hospitalization. As a result, the database is particularly well-suited for evaluating complications and short-term clinical outcomes during inpatient care. In studies on patients with malignancies, the DPC database has been used to evaluate treatment effectiveness and adverse drug reactions associated with anticancer therapies in routine clinical practice. For instance, a study comparing infection risks associated with docetaxel and afatinib therapy in patients with lung cancer demonstrated that respiratory infections occurred significantly more frequently in the docetaxel group, whereas no significant difference was observed in urinary tract infections [46]. Multivariate analyses further showed that docetaxel therapy was significantly associated with the occurrence of respiratory infections compared to afatinib therapy. In randomized controlled trials, the classification and incidence of infectious complications have not always been fully characterized [47]. By contrast, analyses using DPC data can reveal differences in infection risk under real-world clinical conditions. Notably, these findings are consistent with experimental studies suggesting that docetaxel suppresses ciliary beat frequency in ciliated airway cells, potentially impairing mucociliary clearance, and thereby increasing susceptibility to respiratory infections [48]. Thus, the findings from the DPC-based analyses were consistent with the mechanistic insights from the experimental studies.

The DPC database was used to evaluate the impact of healthcare systems and clinical interventions on patient outcomes. In the field of infectious diseases, several studies have evaluated the association between pharmacist-led interventions or the involvement of infection control teams and the safety and efficacy of antibiotics [49, 50]. These studies used reimbursement claims, such as drug management and guidance fees, and infection prevention and control premiums, as proxy indicators of clinical interventions by pharmacists and infection control teams. These analyses highlighted the important roles of pharmacists and multidisciplinary healthcare teams in optimizing pharmacotherapy. Similar research evaluating the impact of pharmacist and multidisciplinary team interventions on treatment outcomes and adverse drug reactions should be further promoted in the field of cancer-related research.

Taken together, the DPC database provides a valuable resource for evaluating short-term clinical outcomes and adverse drug reactions in hospitalized patients. However, because the DPC database primarily captures inpatient care, it has inherent limitations when evaluating outpatient management and long-term patient follow-up. These characteristics should be considered when interpreting the findings derived from DPC-based analyses.

## Future perspectives

The RWDs have become an increasingly important resource for evaluating drug safety and outcomes in cancer-related clinical practice. As highlighted in this review, different types of RWD have distinct methodological strengths and limitations. Therefore, selecting appropriate data sources according to specific research questions is essential for generating reliable clinical evidence. In addition, combining multiple RWD sources in a complementary manner may further enhance the robustness and interpretability of the findings by leveraging the strengths of each data type.

With the ongoing advances in health information technology, future RWD are expected to incorporate increasingly detailed clinical information, including laboratory data, genomic profiles, and patient-reported outcomes. The integration of multidimensional data with insights from basic and data sciences may enable a more precise evaluation and prediction of adverse drug reactions. These developments have the potential to generate high-quality patient-centered evidence and further advance pharmaceutical science research in cancer-related fields, ultimately contributing to safer and more effective pharmacotherapies in real-world clinical practice.

## Data Availability

No datasets were generated or analysed during the current study.

## Abbreviations

AKI: Acute kidney injury
bDMARDs: Biological disease-modifying antirheumatic drugs
CMV: Cytomegalovirus
DPC: Diagnosis Procedure Combination
EMR: Electronic medical records
HSCT: Hematopoietic stem cell transplantation
MTX: Methotrexate
RDI: Relative dose intensity
RWD: Real-world data
